# Mental Health Status of Indian Migrant Workers in the United Arab Emirates during the COVID-19 Pandemic

**DOI:** 10.3390/healthcare11111554

**Published:** 2023-05-25

**Authors:** Md Imran Khan, Mohammed Arshad Khan, Noorjahan Sherfudeen, Asheref Illiyan, Mohammad Athar Ali

**Affiliations:** 1 Department of Economics, Jamia Millia Islamia (A Central University), New Delhi 110025, India; 2 Accounting Department, College of Administrative and Financial Sciences, Saudi Electronic University, Riyadh 11673, Saudi Arabia; 3 Department of Finance, College of Administrative and Financial Sciences, Saudi Electronic University, Riyadh 11673, Saudi Arabia

**Keywords:** COVID-19 pandemic, migrant workers, mental health, psychological impact, UAE, Bihar, Uttar Pradesh, labour migration

## Abstract

Migration has become a de facto phenomenon in the contemporary globalized world and India is not untouched. Indian labourers from the states of Bihar and Uttar Pradesh migrated to the UAE in search of better jobs and prospects. They migrated alone and left behind their families. The distance between them and their family can also create mental disorders; therefore, it becomes necessary to analyze the mental health of the migrant workers during the COVID-19 pandemic. The current study is quantitative and based on a sample survey approach. The researchers collected 416 samples through a structured questionnaire and used the snowball sampling technique. Descriptive statistics, Pearson’s correlation coefficient, chi-square test and logistic regression were utilized to analyze and interpret the results. The outbreak of coronavirus disturbed their livelihood resulting in a cut to their salary or earnings; in total, 83% of migrants were affected by the COVID-19 outbreak in terms of loss of their income, out of which 76% were affected by less than AED 1000. The respondents’ mental health was worrisome, but they were hopeful for the future. In total, 73.5% of respondents felt nervous, 62% felt depressed, 77% felt lonely, 63.4% had a hard time sleeping, and 63% had difficulties concentrating. The findings of the study draw attention to the policymakers to carry out necessary provisions to the targeted psychologically affected community. The findings also suggest creating awareness among the people by using social networking sites and diagnosing mental disorders on an urgent basis.

## 1. Introduction

Migration has become a de facto phenomenon in the contemporary globalized world and India is not untouched. Based on the results of the 2011 census, it was estimated that 37 per cent of India’s population is made up of migrants. There is a substantial amount of migration from India to other countries too. The survey estimates that 18 million individuals reside outside of India [1]. The Indian diaspora is the largest diaspora according to the Population Division of the UN Department of Economic and Social Affairs study titled International Migration 2020 [2,3]. According to MEA data, over 3.5 million Indians are living in the UAE, making up the biggest group and almost 30% of the country’s total population (Indian Community in UAE, Embassy of India). Most of them are from Uttar Pradesh, Bihar, and Kerala. In total, 15% of the diaspora in the United Arab Emirates resides in Abu Dhabi, including Dubai. When oil prices spiked in the Gulf in 1973, oil-producing countries began investing heavily in their infrastructure [4]. It led to a substantial influx of migrants in this area because of the necessity for a workforce. After Kerala, the other two Indian states having the highest migration burden are Uttar Pradesh and Bihar [5]. There are varieties of socioeconomic traits that both states share with each other because agriculture is the backbone of both states’ economies, but not enough physical infrastructure exists to accommodate the growing number of available jobs, and also due to the prevalence of low- and moderate-skilled labourers in both states. [6]. People from this area move elsewhere in pursuit of thriving economic security and better future opportunities. Since the 1970s, semi-skilled or unskilled labourers have been in high demand in the UAE, but with time, white-collar workers were also required. However, the workforce in the UAE consists of 70% semi-skilled or unskilled labourers compared to 30% skilled and white-collar workers. This is one of the reasons that migration is on the rise from the above-mentioned Indian regions [7].

The COVID-19 pandemic started in China (Wuhan city) in December 2019, and the World Health Organization (WHO) first declared it a public health emergency on 30 January 2020. However, after the virus spread to 110 countries and the number of cases reached 118,000, the WHO then declared it a global pandemic (11 March 2020). The coronavirus has quickly spread around the globe, resulting in a large number of individuals becoming sick and several deaths [8]. The newly discovered coronavirus made us reconsider the world’s numerous medical institutions. The quick spread of this virus from human to human also led to an economic catastrophe, the collapse of the labour market, and a global health disaster. To stem the spread of the virus, several nations throughout the world imposed a total lockdown and limited travel for their citizens. However, this disrupted not only peoples’ daily lives but also their sources of income [9,10].

For the first time in recent history, the continuing pandemic had reduced the fresh flow of international migration and increased the number of returnee migrants [11]. The World Bank estimates that between 50,000 and 60,000 people from urban India moved to rural areas because of the lockdown’s impact on their safety and economic stability. It also estimates that India brought back 700,000 of its migrants residing in the diaspora with the help of international ships and a special flying programme (Vande Bharat Mission) [3]. During the epidemic, Kerala, a state in southern India, saw the most returnee migrants (about 1.2 million). The UAE is a significant trading partner of India, but the biggest drop in remittances (17%) came from the UAE in 2020, while the overall drop in remittances was only 0.2%. However, India saw an increase in remittances of 8% in 2021 (USD 89.4 billion), which accounts for 3% of its GDP and is expected to grow by around 5% in 2022–2023. Since 2008, India has maintained its position as the world’s top recipient of remittances; on average, Indian migrants from the Gulf nations send USD 396 per month to their homes [12,13]. Indian migrant workers in the United Arab Emirates may have seen a big change in their mental health because of the COVID-19 pandemic. During the pandemic, many of these workers had to deal with situations such as isolation, fear, and uncertainty, which might severely affect their mental health. Apart from this, many migrant workers could not go home to see their families because of the travel restrictions and lockdowns. The longingness of migrants for their family and friends made them feel lonely and alone, which can have a severe effect on their mental health. Additionally, many workers were afraid of losing their jobs or possible instability of supporting their families, which could cause anxiety and stress. The pandemic also threw workers off their normal schedules, making it hard for them to keep up healthy physical and mental habits such as exercise and socializing. These changes can also make people feel stressed out and anxious. Many people have low-paying jobs with bad working conditions, which can make them feel stressed, anxious, or depressed [14]. Therefore, it becomes necessary for us to analyze the mental health of Indian migrant workers. Furthermore, the lack of access to mental health services and support systems can exacerbate the situation. It is crucial to address the mental health needs of these workers to ensure their overall well-being and productivity.

## 2. Review of the Literature

On 29 January 2020, a Chinese family travelling through the UAE reported having contracted the coronavirus; the first confirmed case of the virus had been reported in Wuhan, China, in December of the previous year. The Ministry of Health and Prevention and the UAE government responded and took the necessary steps to stop the coronavirus from spreading. On 8 March 2020, the government started to transition the teaching–learning process online and close the offline classrooms in schools and institutions. As soon as the World Health Organization designated the coronavirus a worldwide pandemic, the central government of the UAE began taking stringent action to stop its spread (11 March 2020). Flights were suspended on 25 March 2020, and restrictions on travel inside the nation and a night-time curfew were also enforced the following day, on 26 March. The night curfew was maintained despite the 4 April 2020 nationwide lockdown, which lasted until the 23 [13]. When people experience a shift in their surroundings, they often feel anxious and vulnerable. When the cause, course, or effects of an outbreak of an infectious disease are unknown, panic and rumours tend to spread [15]. The need to quarantine a large number of people to stop the spread of the virus caused widespread panic among the general population [16]. The psychological and economic fallout from the pandemic has been the subject of several studies. Findings from across the globe demonstrated that most Chinese people, ranging from 7% to 53.8%, had some form of psychological distress during the early stages of the COVID-19 pandemic [16,17,18,19]. Many studies demonstrate that healthcare professionals had greater rates of moderate to severe stress and psychological distress than non-healthcare employees [20,21]. Therefore, people who may be at risk for mental health issues during COVID-19 are frontline employees, including police and hospital workers [22]. We are aware that the incidence of anxiety experienced by people dramatically increased when the pandemic outbreak took place. For instance, in Hong Kong, almost 70% of residents reported anxiety [23]. The study reported that the psychological impact on children and adults in the UAE was moderate to severe. General anxiety disorder had a prevalence of 71% in the general population, with the highest levels of anxiety being reported by younger persons (59.8%) and females (51.7%). The highest percentage of emotional issues in children was reported by parents who had previously worked as educators (26.7%) [24]. The COVID-19 outbreak had big effects on the minds of children as well. They deal with fears, unknowns, big changes in their routines, physical and social isolation, and a lot of stress from their parents [25]. The speed at which the disease spreads and the frequency of emotional distress and social disorder both during and after the outbreak have a substantial impact on the population’s mental states, according to an insightful study that attempted to predict these responses. Despite this, enough resources are frequently lacking to control or lessen the negative consequences of pandemics on psychological well-being and mental health. It is well recognized that psychological variables have a significant impact on how individuals adhere to public health interventions (such as vaccination) and deal with the risk of infection and the ensuing losses. In the first place, it should be acknowledged that, regardless of the normal order of things, persons with an established mental condition have a shorter lifespan and worse results for their physical health than the overall population. The chance of contracting COVID-19, the difficulty of getting tested and treatment, and the adverse physical and psychological repercussions of the pandemic are all enhanced in those with pre-existing mental disorders and substance use issues [26]. Discrimination might arise from the anxiety and fear of infection. As a result of the media’s focus on the COVID-19 outbreak in Wuhan, the entire Chinese population has been unfairly demonized abroad, with labels such as “China virus” and “Wuhan virus” being used to refer this disease [14]. Migrants have been hit worse by the COVID-19 pandemic globally. Several reports have been published indicating their worsening mental well-being [27]. The study conducted on Ethiopian returnees reveals that 55% of the respondents to the survey experienced depressive symptoms; around 48.9% had stress symptoms and 35.6% had stress. The identified causes associated with it were fear of discrimination, experiencing COVID-19-like symptoms, or having no plan as to what to do after the quarantine [28]. According to another study, migrants in the Middle East are more likely to experience anxiety, depression, and suicidal thoughts. In such a situation, the COVID-19 outbreak could expeditiously be exacerbating the psychological distress among the migrated population [29]. One study on the mental distress of migrants from Kerala to the Middle East was conducted. According to their result, the sample size of the total respondents of the online survey was 96, out of which the percentage of clinically severe anxiety levels was 52% and the percentage of chronic depression levels was 41%. The indicators for these symptoms were associated with the level of concern with air traffic restriction [30]. In several media reports, the suffering of the migrants was raised. According to The Hindu’s report, in some cases, the migrants had been denied their wages by their employers and, consequently, they had no other alternative left; they had to either return to their homes or, if could not return, they had to depend upon money being sent from their homes. Given the scenario, their identification of this experience was that they felt lonely in a foreign land [31]. Another study illustrates the psychological impact of COVID-19 on the various ages, gender, and earning groups of returnee migrants from Saudi Arabia. The result of the study illustrates that the psychological disturbance caused by COVID-19 could be traced across all genders, ages, and earning groups of migrants who returned from Saudi Arabia, but women and people of 51 plus age and high earning groups have been hit severely [32]. Another study was conducted to elaborate on the economic impact of Indian migrant workers in Saudi Arabia, who reported a loss in income and remittances, and several migrants had quit their jobs to take care of their family members in their homeland; however, it is interesting to note that undocumented migrants were affected the most during the pandemic [33,34]. The only study which was conducted to measure the psychological impact on the migrant workers from Bihar and Uttar Pradesh in Saudi Arabia reported a moderate to severe psychological impact [35]. Many studies have been conducted that deal with the problems and prospects of the COVID-19 pandemic. However, very limited studies were found that dealt with the problems and prospects of migrant workers, especially blue-collar workers. This is a significant gap in the literature since migrant workers are among the most vulnerable groups during this pandemic, and their experiences and challenges need to be addressed to ensure their well-being and safety [36]. Therefore, there is a pressing need for more research on the impact of COVID-19 on migrant workers, particularly those in blue-collar jobs.

## 3. Research Gap

The scope of the previous studies indicates that most of the studies on the impact of COVID-19 lack the migrant perspective. Those studies focused on the impact of COVID-19 on migrants are economically oriented only and very few studies have focused on the psychological impact of COVID-19 on migrants. A few studies on the psychological impact have studied those migrants who have returned home, and most of them are Kerala residents. The scope of this proposed study is how COVID-19 psychologically impacted those migrants who are northern Indians from Bihar and Uttar Pradesh who are also still residing in UAE.

## 4. Objectives

1. To explore the impact of coronavirus on salary and remittance reduction of Indian migrant workers working in the UAE.

2. To analyze the mental health of the Indian migrant workers in UAE during the coronavirus pandemic.

## 5. Hypotheses

The current study will test the following null hypotheses.

1. “There is no significant difference in the mental health of the migrant workers across different age groups”.

2. “There is no significant difference in the mental health of the migrant workers across the domicile of Bihar and Uttar Pradesh”.

3. “There is no significant difference in the mental health of the migrant workers across the different number of dependent persons”.

## 6. Research Methodology

The present study analyzes the mental health of Indian migrant workers in the United Arab Emirates during coronavirus diseases. The examination was quantitative in nature and the results are based on a sample survey approach. The researchers drafted the questionnaire following the “General Health Questionnaire-8” and distributed it to the respondents through email and social networking sites such as Twitter, Telegram, and WhatsApp [37]. The sample was collected by using the snowball sampling technique in the month of May 2021 [38]. The structured questionnaire was in the English language and included two sections. In Section 1, the questionnaire related to the demographic information of the migrants such as current age, the highest level of education, religious belief, monthly income, number of dependents, remittance, and loss of income during the pandemic. The questionnaire related to the mental health of the migrants in Section 2. The researchers included six important questions that provided the basis for the analysis of the mental health of the migrants. The instruction was given to the respondents and the questionnaire was requested to be filled out by only Indian male migrants who belong to the Indian states of Bihar and Uttar Pradesh. The major reason that the researcher had chosen a sample of migrant workers from Uttar Pradesh and Bihar was that most of the semi-skilled and unskilled workers were supplied by these states only. The blue-collar workers were working for their survival with a very low level of income. These were the working groups neglected by the authority during the pandemic. Therefore, their mental health needed to be checked. The study aimed to investigate the impact of the pandemic on the mental health of these migrant workers who were already struggling with financial and social challenges. The findings could inform policymakers to develop interventions that address the mental health needs of this vulnerable population. The snowball sampling method gave the researcher a chance to talk to the respondents. A detailed telephonic interview was conducted with the migrant workers who were not comfortable understanding the English language and unaware of the use of a Google Forms questionnaire. The telephonic interview helped the researcher to establish a personal connection with the respondents and gain their trust, which would have been difficult to achieve through an online questionnaire. Additionally, this method allowed for more in-depth responses and clarification of any misunderstandings. Of the 720 Indian migrant labourers who were sent the link to a Google Form questionnaire, the researchers were able to successfully collect 416 replies that were properly filled out. The data from the collected sample, which consisted of 416 Indian migrant workers, were analyzed with the help of version 28 of the Statistical Package for Social Science (IBM, Chicago, IL, USA) [39]. The study also uses secondary data collected through different research articles, reports, and websites. Descriptive statistics, Pearson’s correlation coefficient, chi-square test, and logistic regression were utilized to analyze and interpret the results.

## 7. Results and Discussion

### 7.1. Demographic Profile of the Indian Migrant Workers in the UAE

The demographic profile of the sampled migrant workers from the Indian states of Uttar Pradesh and Bihar who are working in the United Arab Emirates is presented in Table 1. The demographic data help us to understand the socio-cultural scenario of the given situation. In this context, the demographic details give a proper account of the background of the respondents to inquire about the causes and consequences. According to the table, the total number of migrants who were interviewed was 416. Out of that number, approximately 52% (216 respondents) were from Uttar Pradesh, while the remaining 48% (200 respondents) were from Bihar. Among these respondents, the percentage of respondents under the age of 40 was 51%, with 128 respondents coming from the state of Bihar and 88 respondents coming from the state of Uttar Pradesh. On the other hand, 47% were over the age of 40. Of these, 72 were from the state of Bihar and 128 were from the state of Uttar Pradesh. As a result, we can conclude that most migrant workers fall into the younger age bracket.

Most migrant workers adhere to Islam (89.42%), while the remaining 10.58 per cent are followers of Hinduism. Both Uttar Pradesh and Bihar, which are states in India, have a relatively low level of educational attainment [40]. As a direct consequence of this, the educational standing of the migrant worker is regarded as being of a low standard. It is believed that approximately 10% of all migrant workers are illiterate and that more than 12% of migrant workers have not completed any type of formal education. It is important to note that over thirty per cent of them hold a bachelor’s degree, and only three percent hold a master’s degree. Based on these findings, we can conclude that most of the migrants included in the sample were under the age of 30, predominantly adhered to Islam, and had low formal education.

The migration has helped not only the people who moved and their families but also the economy of the state. The money that migrants sent home has been a big part of how the economy of that area has grown [41]. The presented data pertain to the monthly remittances that migrant workers send to their families, as well as their monthly earnings in the United Arab Emirates, as measured in Arab Emirates Dirhams (AED). The earnings range from AED 1500 to 2500, which accounts for approximately 43% of the total respondents. Additionally, 30% of respondents had a monthly income that was greater than AED 2500, while 23% of respondents had monthly earnings that were lower than AED 1500. It also reveals that fifty percent of the people who responded sent more than AED 1000 with their remittances, while the other fifty percent contributed less than AED 1000. It is interesting to note that the data indicate that migrants from Bihar send a larger percentage of their monthly earnings back home as remittances than migrants from Uttar Pradesh do, as 72 out of 216 respondents from Uttar Pradesh are sending remittances that are less than 1000 rupees (AED).

The duration of work of migrant workers is an important issue that needs to be addressed, as it can have significant impacts on both the workers themselves and the countries that employ them. Most respondents had between four and eight years of experience, which accounts for nearly half of the total number of respondents, whereas the number of respondents with below four years of experience was 26.4% and the number of respondents with above eight years of experience was 24.5%.

The occupations of migrant workers have been divided into three categories: blue-collar, white-collar, professionals and businessmen. The results demonstrate that most migrants have a blue-collar profession, which accounts for 63% of all migrants, while 31% of migrants have a white-collar profession, and only 6% are businessmen and professionals. Blue-collar jobs are those that may entail physical labour, such as construction or landscaping. Semi-skilled or unskilled labourers may be required for tasks such as mining, driving, construction, recycling, and domestic help, while white-collar professions require skills and expertise. They involve clerical and official setups, and they get paid better than blue-collar jobs [42]. The data depict that most of the migrants are either unskilled or semi-skilled. As a result of this, we are led to infer that most migrant workers who have been employed in the UAE for longer than four years are working in low-skilled positions.

### 7.2. Spread of Coronavirus and Loss in Income

The spread of the coronavirus (COVID-19) has had a big effect on economies around the world, causing many people to lose their jobs and make less money. Because of the pandemic, many businesses have had to close down, either temporarily or permanently. This has caused people to lose their jobs in many different fields, including tourism, hospitality, and retail. People who have lost their jobs have seen a big drop in their income, which has put many households in a tough financial situation. The pandemic has made it harder for people who still have jobs to make as much money as they could. Many businesses have had to cut back on operations or hours, which has led to less pay for workers. Others have had to find ways to work from home, which could mean less pay or more expenses for the employees. Furthermore, the economic uncertainty caused by the pandemic has made it difficult for people to plan for their financial future, such as saving for retirement or investing in their education. The situation has highlighted the need for more support and resources to help individuals and families navigate these challenging times. After the COVID-19 epidemic in 2019 [10], migrants had a major drop in their income and were confronted with several challenges, including the loss of their jobs, a decrease in their income, increased working hours without a corresponding gain in pay, and many more [43]. According to Table 2, it appears that 83 percent of migrants saw a reduction in their income as a direct result of the COVID-19 pandemic. Of those affected, 76% had losses that were less than AED 1000, while just 7% had losses that were greater than AED 1000. Alterations to one’s employment status and subsequent financial setbacks are bound to have psychological consequences in addition to their financial ones. It is important to consider the mental health implications of such financial losses, particularly for vulnerable populations such as migrants who may already be experiencing stresses related to displacement and resettlement. Providing support and resources for coping with these challenges can help mitigate their negative impact on mental health.

### 7.3. Mental Health of the Indian Migrant Workers in UAE during Coronavirus

The COVID-19 outbreak did not just affect the migrants financially, but it impacted them psychologically too. The worries of an uncertain future while losing jobs, being quarantined in a foreign land, and returning in an emergency without any job security were all quite enigmatic and nauseating. The pandemic has also exposed the vulnerabilities of the migrant workforce and highlighted the need for better protection and support for them, especially during times of crisis [44]. Table 3 illustrates the mental health of the migrants. The respondents’ mental health was worrisome, but they were hopeful for the future. In total, 73.5% of respondents felt nervous, 62% felt depressed, 77% felt lonely, 63.4% had a hard time sleeping, and 63% had difficulties concentrating. However, 89% of respondents were quite hopeful that all this had to end and that they had a future. The data show us that the respondents belonging to Uttar Pradesh were more worrisome and had more grave conditions than respondents from Bihar; more than 60% of respondents belonged to Uttar Pradesh, and those who responded yes to the statements, stated their worsening mental effects, while only 55% of respondents from Uttar Pradesh were hopeful. This could have been caused by various concerns such as the well-being of their family members back at home because of the severe COVID-19 situation in their home state Uttar Pradesh. According to media reports, Uttar Pradesh was facing a much more severe COVID-19 outbreak than Bihar. This highlights the need for targeted mental health support for those from Uttar Pradesh, especially those with family members affected by COVID-19. It also emphasizes the importance of addressing the COVID-19 situation in Uttar Pradesh to alleviate the mental health burden on its residents.

The Pearson correlation coefficient is widely used to measure the linear correlation between the variables. The value of the correlation coefficient lies between −1 and +1. A negative value represents a negative correlation between the variables whereas a positive value represents a positive correlation between the variables [45]. Correlation coefficients were used to determine whether there was any linear correlation between the mental health of migrant workers and age, level of education, and number of family members.

Table 4 shows the result of the correlation coefficient between the mental health of the migrants and age, level of education, and the number of members in their respective families. The coronavirus pandemic disturbed the mental health of the migrants. It was found that the majority of the migrant workers were feeling nervous. The nervousness among the migrant workers shows their fear and anxiety due to the high transmission of the deadly coronavirus disease. The correlation coefficient result shows a negative low correlation (r = −0.164 and *p*-value *=* 0.018) between age and feeling nervous and was found to be statistically significant, whereas the correlation coefficient between feeling nervous and the level of education was found to be positive (r = 0.192, *p*-value = 0.062) but statistically insignificant. The researchers also tried to find the correlation between feeling nervous and the number of dependents (size of family) on the migrant’s earnings. The result confirms the positive correlation between feeling nervous and the number of dependents (r = 0.302, *p* < 0.05). The higher the number of dependents, the more nervous the migrant workers will be.

Depression among Indian migrant workers in the UAE was common during the lockdown period of the pandemic. Depression is a type of mood disorder that results in loss of interest and feelings of sadness. It can change mood and physical health. A drop in migrant workers’ productivity is directly correlated with their prevalence of depression. This research tries to establish the relationship between feeling depressed and the age of a migrant worker, which is found to be negative and statistically significant (r = −0.215, *p* < 0.05), whereas feeling depressed was positively related to the level of education of the migrants but statistically insignificant. The prevalence of feeling depression was positively related to the number of dependents (size of family) (r = 0.160, *p* < 0.05).

Male migrants from Bihar and Uttar Pradesh to the Gulf countries left their family in their homeland, resulting in the feeling of loneliness. This can be defined as unpleasant emotions in people. The researchers established the relationship between felt loneliness and the age of the migrant, which was found to be negative and statistically significant (r = −0.154, *p* < 0.05), whereas loneliness is positively related to the level of education but found to be statistically insignificant (r = 0.084, *p* > 0.05). There was a statistically significant negative correlation between feeling lonely and the number of dependent people (r = −0.152, *p* < 0.05).

Hopefulness about the future is one of the important parameters to understanding the mental health status of people. The meaning of hopeful is “optimism about a future event”. The researchers also tried to establish the relationship between hope about the future and the age and level of education of the migrants, which was found to be negative (r = −0.213, *p* < 0.05) but statistically insignificant; however, the relationship between the number of dependents was positively related with feeling hopeful and statistically significant (r = 0.251, *p* < 0.05).

Sleeping difficulties were another important parameter in understanding the mental health of the migrant worker and it is interesting to know how they are related to different variables. A hard time sleeping may cause an inability to focus and irritation. A hard time sleeping was negatively related to the age of the migrant (r = −0.208, *p* < 0.05) and found to be statistically significant, whereas it was positively related to the level of education of the migrant workers (r = 0.057) but statistically insignificant. Similarly, it was found that a hard time sleeping was positively related to the number of dependents (r = 0.315) and found to be statically significant at a 5% level.

The researchers also asked the respondents about concentration problems during the lockdown period of the coronavirus pandemic and tried to establish the relationship between age, education, and the number of dependents. The Pearson correlation coefficient result confirms that it was negatively related to the age of the migrants and found to be statistically significant (r = −0.229, *p* < 0.05). However, the relationship between the level of education and concentration problems was also negative but statistically insignificant. A moderate positive correlation was found between difficulties in concentration and the number of dependents (r = 0.400, *p* < 0.05).

### 7.4. Age, Place of Origin, Family Members and Mental Health of the Migrant Workers

A chi-square test was utilized to discover the association between the mental health of migrant workers across the different age groups [46]. The basic idea behind the use of the chi-square test was to compare observed results with the expected result. To test the null hypothesis of “there was no significant difference in the mental health of the migrant workers across different age groups”, a chi-square test was applied. Migrant workers were grouped into three categories according to age and compared to their mental status.

The results in Table 5 show that there is a statistically significant difference in feeling depressed, feeling nervous, feeling lonely, having a hard time sleeping, and having difficulty concentrating among different age groups at a 5% level of significance; however, hope about the future was statistically insignificant in different amongst age groups at a 5% level of significance. Therefore, we reject the null hypothesis. This means that there was a significant difference in the mental health of migrant workers among the different age groups. The test results and descriptive data in Table 5 suggest that the migrant workers’ mental health deteriorates from moderate to severe with age. The higher the age of the migrant, the greater the number of duties and the greater the economic strain, both of which bring about a severe deterioration in their mental condition [47]. As a consequence of this, we are able to arrive at the conclusion, on the basis of the empirical evidence that is shown in the table, that the mental health of migrant workers who were of a greater age was in critical condition.

This part of the research was conducted with the intention of demonstrating that a migrant worker’s mental health is affected not only by the conditions that are prevalent in the country in which they are working but also by the conditions that are prevalent in the country from which they belong, i.e., where their loved ones live. In comparison to Bihar, the number of people who died from COVID-19 was far higher in Uttar Pradesh [35]. A chi-square test was utilized to discover the association between the mental health of migrant workers and the different states to which the employees belong [45]. To test the null hypothesis of “there was no significant difference in the mental health of the migrant across different domiciles”, a chi-square test was applied. The result in Table 6 shows that the relationship between the variables was found to be statistically significant as shown by the chi-square value and the *p*-value, which is less than 0.05 (5% level of significance). Therefore, we reject the null hypothesis; it simply means that there are significant differences in the mental health of Indian migrant workers across the country. This is simply because the prevailing situation in each district was different.

Several studies have revealed that the transmission of coronavirus has altered occupational status, earnings, working conditions, and other aspects of employment [48]. Consequently, migrant workers who have a larger number of family members to support are likely to be more anxious than those who have a smaller number of family members. The researcher tried to establish a significant association between the mental health of the migrant worker and the number of family members; to test the null hypothesis of “there was no significant difference in the mental health of the migrant workers across the different number of dependent persons”, a chi-square test was applied. The results of the chi-square test reject the null hypothesis in all parameters of measuring mental health except feeling lonely, as the *p*-value is less than 0.05 (5% level of significance). The migrant worker may feel less isolated during the lockdown if they can maintain phone contact with more of their dependents than usual. However, in all other cases, mental health among migrant workers is high, with a higher number of dependent persons. This means that there is a significant difference in the mental health of migrant workers depending on the different numbers of dependent persons.

### 7.5. Determinants of Mental Health: A Logistic Regression

A logistic regression model, which is a type of statistical analysis, was used in order to make an estimate of the extent to which various factors influence the mental health of migrant workers. The dependent variable of mental health is a dichotomous variable, with the possible outcomes being either good mental health during coronavirus or no such outcome. If the probability of an event is *p*, then the odds of having good mental health can be calculated as the ratio of the probabilities of having good mental health and not having good mental health, which is written as *P*/(1 − *P*). The dependent variable in the equation was the participant’s mental health, which was obtained from six indicators measuring mental health: being depressed, being nervous, being lonely, having a hard time sleeping, having difficulties concentrating, and being hopeful about the future. The dependent variable mental health (Yi) = number of options chosen/total number of options. The predictors in the equation were the participant’s age, domicile, educational qualification, and other sources of income. Table 7 shows the result of the logistic regression. The age of the migrant worker was one of the significant predictors in the equation that impacts the mental health of migrant workers. This indicates that deterioration in mental health is likely to occur with increasing age. In a similar vein, the fact that the number of dependent people was another predictor that had an influence on the mental health of migrant workers suggests that a migrant worker’s mental health will deteriorate as their number of responsibilities increases. The researchers tried to test another hypothesis: that the mental health of migrant workers differs with different levels of education. The result rejects the null hypothesis, as the mental health of the migrant workers differs with respect to their level of education. The more you know and understand about the spread of and deaths from coronavirus, the more your mental health will be disturbed. The mental health of migrant workers was not only disturbed by the prevailing conditions in their destination country but was also influenced by the prevailing situation in their native states. The domiciles of migrant workers were one of the significant predictors of their mental health. Therefore, we can conclude that the mental health of the migrant workers from Uttar Pradesh was more severely affected than that of the migrants from Bihar. The researchers asked the respondent about the other sources of income for their family besides remittances. The results of the logistic regression confirm that the better the sources of income, the better the mental health of the migrant workers. Overall, this study highlights the importance of considering various factors that can impact the mental health of migrant workers. It also emphasizes the need for policies and interventions that address these factors to improve the mental well-being of this vulnerable population.

## 8. Limitations

The study has several limitations. The current study focuses on Indian male migrant workers living in the United Arab Emirates during the coronavirus pandemic. The study does not include a sample of migrant workers from outside of India. The results are based on a small sample size (416) and use few tests and techniques. The researchers utilized descriptive statistics, a correlation coefficient, and a chi-square test to interpret the results; however, one can use other models and test to validate the study. The researchers did not include a sample of female migrant workers, students, or children. Migrants from Indian states other than Bihar and Uttar Pradesh were excluded from the research. The mental health of the migrant workers was analyzed by using a few parameters such as nervousness, depression, loneliness, sleeping problems, and concentration problems. The limitations of the study define the scope for further studies. One can examine the psychological impact by using other parameters of measuring mental health which are not included in the study. In addition to the mental health of female migrant workers, students and the children of migrant workers can also provide more opportunities for the examination of the psychological impact. The sample was collected by using a non-random sampling technique (snowball) during the early stage of the pandemic; therefore, it may be possible that the mental health of migrants worker could change in the long term, and there is scope for further study on the same research group.

## 9. Policy Implications

The findings and the results of the sample study draw the attention of concerned officials and policymakers in order that they carry out necessary interventions for the targeted psychologically affected community. The study also recommends creating awareness among people by using social networking sites and diagnosing mental disorders on an urgent basis. The government should make arrangements for counsellors and psychiatrists with free consultations with easy access. The government of the United Arab Emirates should also direct industries that employ international migrants to hire counsellors. To create awareness among the community on how to deal and behave with a person with a mental disorder, the government should take the initiative to form social groups along with social reformers and influence people at the panchayat level. It is also crucial to ensure that screening and intervention for psychosocial concerns be delivered in healthcare settings because many COVID-19 cases will be recognized and treated by staff with less or no expertise in mental health. Therefore, it is important that those in charge of the healthcare system, as well as first responders and healthcare professionals, receive education and training on psychological issues. These measures will help in reducing mental disorders among migrant workers.

## 10. Conclusions

The outbreak of the pandemic was a medical emergency; therefore, it become necessary to analyze the mental health of the people. Indian migrant labourers from the states of Bihar and Uttar Pradesh migrated to the United Arab Emirates in search of better jobs and prospects. They migrated alone and left behind their families in their homeland. The distance from their family can also create mental disorders; therefore, it became necessary for researchers to analyze the mental health of migrant workers. The current study attempted to examine the mental health of Indian migrant workers living in the UAE during the lockdown period of the pandemic. The study was quantitative, and the examination of the result was based on a sample survey approach. The researchers collected 416 samples of migrant labour through a structured questionnaire and used the non-probability snowball sampling technique. The collected sample data of 416 Indian migrant workers were scrutinized by using the ‘Statistical Package for Social Science’ version 28. Descriptive statistics, Pearson’s correlation coefficient, a chi-square test and logistic regression were utilized to analyze and interpret the results. The demographic profile of the migrant labour reveals that most of the labourers (52% below the age of 40) are of a young age group and religious background of Islam (89.42%). More than 20% of the sample data was either illiterate or able to read and write only. The monthly earnings of most of the migrants (66%) were below AED 2500, whereas half of the respondents remitted below AED 1000 per month. The reported job profile of the migrants reveals that most of them migrants have a blue-collar profession which amounts to 63%, while 31% of migrants have a white-collar profession, and only 6% are businessmen and professionals. The outbreak of coronavirus disturbed their livelihood, resulting in a cut to their salary or earnings; in total, 83% of migrants were affected by the COVID-19 outbreak in terms of loss of their income. Out of these, 76% bore a loss of below AED 1000 and only 7% lost more than AED 1000. The respondents’ mental health was worrisome, but they were hopeful for the future. In total, 73.5% of respondents felt nervous, 62% felt depressed, 77% felt lonely, 63.4% had a hard time sleeping, and 63% had difficulties concentrating. The correlation coefficient results also established the relationship between indicators of mental health and age, level of education, and the number of dependent people. The chi-square test results confirm that there is a significant difference between the mental health of migrant workers across different age groups, different levels of education, different domiciles, and different numbers of dependent persons. The logistic regression results rejected the null hypothesis proposed in the study. The current study reports that the mental health of migrant workers was worrisome during the pandemic. The predictors of mental health were age, level of education, number of dependents, domicile, and other sources of income. It was interesting to observe that the older the migrant workers, the worse their mental health was. Additionally, older people will likely experience more deaths and positive COVID-19 test results, according to media reports and research. However, it was complicated to observe how the level of education affected the mental health of the migrant workers. It has always been observed that the more you are aware of a pandemic, the greater your fear of it. Apart from that, the number of people dependent on you also affects your mental health; the more dependents you have, the worse your mental health is. Mental health is not only influenced by the prevailing condition in the host country of the migrant workers but also the prevailing situation in their home country. The findings of this study can be useful in developing interventions and support systems for migrant workers to improve their mental health. It is important to address the factors that contribute to their mental health, including awareness of pandemics and the number of dependents they have.

## Figures and Tables

**Table 1:** Demographic profile.

Age	Bihar	Uttar Pradesh	Total	Percentage
Below 40	128	88	216	51.92%
Above 40	72	128	200	47.08%
Total	200	216	416	100%
Religion	Bihar	Uttar Pradesh	Total	Percentage
Muslim	184	188	372	89.42%
Hindu	16	28	44	10.58%
Total	200	216	416	100%
Level of Education	Bihar	Uttar Pradesh	Total	Percentage
Not Educated	24	14	38	09.14%
Able to read and write	34	20	54	12.98%
10th level	44	22	66	15.87%
12th Level	34	82	116	27.88%
Graduate	60	66	126	30.29%
Post-Graduate	04	12	16	03.84%
Total	200	216	416	100%
Monthly Earning	Bihar	Uttar Pradesh	Total	Percentage
Below 1500 (AED)	50	46	96	23.08%
1500–2500 (AED)	100	80	180	43.27%
Above 2500 (AED)	50	90	140	30.29%
Total	200	216	416	100%
Monthly Remittance	Bihar	Uttar Pradesh	Total	Percentage
Below 1000 (AED)	64	144	208	50.00%
Above 1000 (AED)	136	72	208	50.00%
Total	200	216	416	100%
Working Experience	Bihar	Uttar Pradesh	Total	Percentage
Below 4	24	86	110	26.44%
4–8	98	106	204	49.04%
Above 8	78	24	102	24.52%
Total	200	216	416	100%
Profession/Occupation	Bihar	Uttar Pradesh	Total	Percentage
Blue-collar	146	117	263	63.22%
White-collar	52	76	128	30.77%
Professionals and Businessmen	2	23	25	6.01%
Total	200	216	416	100%

Source: Calculated by authors.

**Table 2:** Loss in income during coronavirus lockdown.

Loss in Income	Bihar	Uttar Pradesh	Total	Percentage
No loss	36	34	70	16.83%
Below 1000 (AED)	158	158	316	75.96%
Above 1000 (AED)	6	24	30	7.21%
Total	200	216	416	100%

Source: Calculated by authors.

**Table 3:** Mental health status of the Indian migrants.

Statement	Variable	Bihar	Uttar Pradesh	Total	Percentage
Felt nervous	Yes	114	192	306	73.56%
	No	86	24	110	26.44%
Felt depressed	Yes	78	180	258	62.02%
	No	122	36	158	37.98%
Felt lonely	Yes	126	194	320	76.92%
	No	74	22	96	32.08%
Felt hopeful	Yes	164	206	370	88.94%
	No	36	10	46	11.06%
Hard time sleeping	Yes	76	188	264	63.46%
	No	124	28	152	36.54%
Difficulties in concentration	Yes	64	198	262	62.98%
	No	136	18	154	37.02%

Source: Calculated by authors.

**Table 4:** Pearson’s correlation coefficient matrix.

Variable	Age Pearson Correlation	*p*-Value
Felt nervous	0.164	0.018
Felt depressed	−0.215	0.002
Felt lonely	−0.154	0.026
Felt hopeful	−0.104	0.136
Hard time sleeping	−0.208	0.003
Difficulties in concentration	−0.229	0.001
Variable	Education Pearson Correlation	*p*-Value
Felt nervous	0.129	0.062
Felt depressed	0.041	0.553
Felt lonely	0.084	0.229
Felt hopeful	−0.088	0.207
Hard time sleeping	0.057	0.416
Difficulties in concentration	−0.051	0.467
Variable	Number of Dependent Pearson Correlation	*p*-Value
Felt nervous	0.302	0.000
Felt depressed	0.160	0.021
Felt lonely	−0.152	0.028
Felt hopeful	0.251	0.000
Hard time sleeping	0.315	0.000
Difficulties in concentration	0.400	0.000

Source: Calculated by authors.

**Table 5:** Mental health and age of migrant workers.

Age.	Felt Nervous	
	No	Yes
Below 30	36.8%	63.2%
30–40	32.9%	67.1%
Above 40	15.3%	84.7%
*X*^2^ = 9.390, *p*-Value = 0.009		
	Felt Depressed	
Below 30	48.2%	51.8%
30–40	39.5%	60.5%
Above 40	27.1%	72.9%
*X*^2^ = 8.135, *p*-Value = 0.017		
	Felt Lonely	
Below 30	30.6%	69.4%
30–40	28.9%	71.1%
Above 40	12.9%	871%
*X*^2^ = 8.359, *p*-Value = 0.015		
	Felt Hopeful About Future	
Below 30	13.2%	86.8%
30–40	11.8%	88.2%
Above 40	9.4%	90.6%
*X*^2^ = 0.448, *p*-Value = 0.799		
	Hard Time Sleeping	
Below 30	44.7%	55.3%
30–40	36.8%	63.2%
Above 40	28.2%	71.8%
*X*^2^ = 06.094, *p*-Value = 0.048		
	Difficulties in Concentration	
Below 30	42.4%	57.6%
30–40	42.1%	57.9%
Above 40	29.4%	70.6%
*X*^2^ = 8.167, *p*-Value = 0.017		

**Table 6:** Place of origin, family members and mental health of migrant workers.

Variable	Domicile Chi-Square Value	*p*-Value
Felt nervous	27.146	0.000
Felt depressed	43.324	0.000
Felt lonely	21.032	0.000
Felt hopeful	09.438	0.002
Hard time sleeping	53.845	0.000
Difficulties in concentration	79.285	0.000
Variable	Number of Dependent Chi-Square Value	*p*-Value
Felt nervous	20.285	0.000
Felt depressed	19.088	0.001
Felt lonely	06.713	0.152
Felt hopeful	17.444	0.002
Hard time sleeping	34.269	0.000
Difficulties in concentration	44.755	0.000

Source: Calculated by authors.

**Table 7:** Logistic regression result.

Logistic Regression Result					
	Exp (B)	S. E	Sig.	95% C.I. for EXP (B) Lower Upper	
Age	1.061	0.018	0.001	1.024	1.099
Domicile	0.331	0.327	0.001	0.175	0.629
Educational Qualification			0.013		
No Formal Education	2.051	0.548	0.190	0.700	6.010
School Level Education	4.026	0.515	0.007	1.469	11.037
Graduate and Postgraduate	4.161	0.464	0.002	1.675	10.340
Doctorate	1.759	0.411	0.170	0.785	3.940
Number of Dependents			0.000		
Below 3	11.011	0.675	0.000	2.932	41.349
4–5	6.011	0.373	0.000	2.893	12.490
Above 5	2.631	0.352	0.006	1.320	5.243
Other Sources of Income	0.358	0.368	0.005	0.174	0.735
Constant	0.231	0.838	0.080		

Source: Calculated by authors, R^2^ = 0.409 (Negelkerke), 0.240 (Hosmer and Lameshow). Yi (Mental Health) = Number of option chosen/total number of options, S.E = Standard error, *n* = 416.

## Data Availability

The data used to support the findings of this study are available from the corresponding author upon request.
